# Chemerin: A Potential Regulator of Inflammation and Metabolism for Chronic Obstructive Pulmonary Disease and Pulmonary Rehabilitation

**DOI:** 10.1155/2020/4574509

**Published:** 2020-04-06

**Authors:** Jian Li, Yufan Lu, Ning Li, Peijun Li, Zhengrong Wang, Wang Ting, Xiaodan Liu, Weibing Wu

**Affiliations:** ^1^School of Kinesiology, Shanghai University of Sport, Shanghai 200438, China; ^2^School of Rehabilitation Science, Shanghai University of Traditional Chinese Medicine, Shanghai 201203, China; ^3^Institute of Rehabilitation Medicine, Shanghai Academy of Traditional Chinese Medicine, Shanghai 201203, China

## Abstract

Chronic obstructive pulmonary disease (COPD) features chronic inflammatory reactions of both intra- and extrapulmonary nature. Moreover, COPD is associated with abnormal glucose and lipid metabolism in patients, which influences the prognosis and chronicity of this disease. Abnormal glucose and lipid metabolism are also closely related to inflammation processes. Further insights into the interactions of inflammation and glucose and lipid metabolism might therefore inspire novel therapeutic interventions to promote lung rehabilitation. Chemerin, as a recently discovered adipokine, has been shown to play a role in inflammatory response and glucose and lipid metabolism in many diseases (including COPD). Chemerin recruits inflammatory cells to sites of inflammation during the early stages of COPD, leading to endothelial barrier dysfunction, early vascular remodeling, and angiogenesis. Moreover, it supports the recruitment of antigen-presenting cells that guide immune cells as part of the body's inflammatory responses. Chemerin also regulates metabolism via activation of its cognate receptors. Glucose homeostasis is affected via effects on insulin secretion and sensitivity, and lipid metabolism is changed by increased transformation of preadipocytes to mature adipocytes through chemerin-binding receptors. Controlling chemerin signaling may be a promising approach to improve various aspects of COPD-related dysfunction. Importantly, several studies indicate that chemerin expression *in vivo* is influenced by exercise. Although available evidence is still limited, therapeutic alterations of chemerin activity may be a promising target of therapeutic approaches aimed at the rehabilitation of COPD patients based on exercises. In conclusion, chemerin plays an essential role in COPD, especially in the inflammatory responses and metabolism, and has a potential to become a target for, and a biomarker of, curative mechanisms underlying exercise-mediated lung rehabilitation.

## 1. Introduction

Chronic obstructive pulmonary disease (COPD) is a systemic disease, not only characterized by an essentially irreversible restriction of airflow [1]. Its pathological process involves both inside and outside the lung. The persistent inflammation in the airway and the destruction of the airway structure are the intrapulmonary pathological manifestations. In addition, a variety of extrapulmonary pathological manifestations on distal organs, the so-called systemic effects and comorbidities of COPD, have been confirmed in patients with COPD [2]. In recent years, both global incidence and mortality of COPD have increased dramatically due to aging in many populations, increased smoking, and deterioration of air quality [3]. COPD is set to become the third leading cause of death and the fifth leading cause of disability in the world before 2030 [4]. Consequently, further insights into the pathogenesis of COPD and the exploration of new targets for COPD treatment may have far-reaching implications for the reduction of the many negative effects of COPD on human health.

Current studies indicate that the pathogenesis of COPD is a complex process [5, 6]. Many factors contribute to the development of intra- and extrapulmonary pathological manifestations in COPD patients. Chronic inflammation existing in the airway is closely related to the occurrence and development of COPD [6]. Pathological intrapulmonary changes in COPD are primarily caused by inflammation in the airways and lungs, in particular by the infiltration of inflammatory cells in large or small airways and lung tissue. Inflammatory mediators released by these cells affect airway remodeling in COPD in multiple ways, thereby impairing the respiratory status of patients. Aside from these pulmonary effects, COPD is associated with abnormalities of extrapulmonary physiological functions, such as skeletal muscle dysfunction, nutritional abnormalities, metabolic complications, osteoporosis, and cardiovascular complications [7]. In turn, metabolic complications, such as glucose and lipid metabolism, have become significant factors underlying the mortality of COPD patients, owing to their influence on pulmonary function and, more generally, the quality of life [8]. Furthermore, the close relationship between abnormal metabolic complications and inflammation has become evident. Disorders of lipid metabolism stimulate inflammation, which further aggravates imbalances in lipid metabolism [9, 10]. Especially for COPD patients, therefore, optimal regulation of inflammation as well as of underlying glucose and lipid metabolism disorders is a key element in the treatment and rehabilitation of COPD.

Chemerin is an adipokine secreted primarily by white adipose tissue and was initially discovered in studies of psoriasis lesions [11]. Subsequent studies demonstrated that chemerin plays a variety of roles in inflammatory and metabolic diseases of the adipose tissue, lung, skin, cardiovascular system, and other organs. Chemerin was used as a proinflammatory or anti-inflammatory regulator to control disease-related inflammatory responses: chemerin promotes the development of inflammation by aggregating different types of inflammatory cells to inflammatory sites in the early stage of inflammation [12, 13]; while at the end of inflammation, the protease released by macrophages (MFs) and apoptotic cells transforms chemerin to play an anti-inflammatory role [14, 15]. Binding chemerin to one of its cognate receptors, chemerin affects adipogenesis and regulates glucose metabolism in adipose tissue: the activation of chemerin promotes the proliferation and differentiation of adipocytes. In addition, the blood supply of adipocytes is also affected by chemerin. Inhibiting the activity of chemerin also inhibits insulin secretion and influences insulin resistance [16, 17]. Recently, the functional characterization of chemerin in COPD has attracted much attention. As an inflammatory chemokine, chemerin supports the inflammatory response of COPD, on the one hand. On the other hand, disordered glucose and lipid metabolism in COPD patients are thought to be under the influence of chemerin. Considering that systemic inflammation and abnormal glucose and lipid metabolism coexist and interact in COPD patients, chemerin's role as a regulator of inflammation and lipid metabolism makes it a potentially highly relevant target for COPD treatment and rehabilitation. At present, the number of reports on chemerin's involvement in COPD is limited, making full elucidation of the role of chemerin in the pathogenesis and treatment of COPD a matter of urgency. The present review summarizes recent advances in this significant area and addresses possible basic mechanisms responsible for them, aimed at providing a reference to guide further analysis of the COPD pathogenesis. In addition, a theoretical framework will be developed for new chemerin-related approaches for the prevention and treatment of COPD.

## 2. Chemerin and Its Receptors

**Figure 1:**
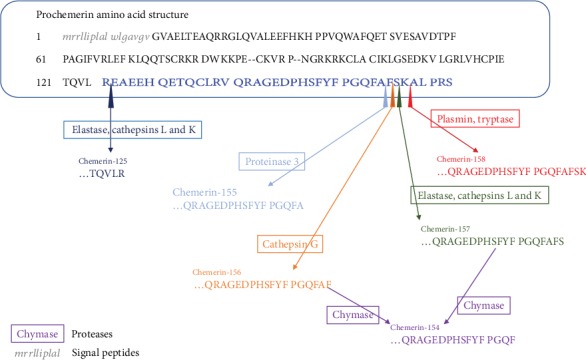
Alignment of preprochemerin amino acid sequence and proteases regulating prochemerin bioactivity. The blue sequence corresponds to the sequence of the potential protease cleavage site of prochemerin.

The structure and function of chemerin and its receptors have been described in detail elsewhere [14, 16] and will be reviewed briefly here. Chemerin was described originally as originating from a precursor protein containing 163 amino acids, whose 20-amino acid hydrophobic peptide was excised from the N-terminal by a protease, producing prochemerin [18, 19]. This preprotein has a low affinity for the chemerin receptors and requires further extracellular C-terminal processing. Under specific conditions, e.g., coagulation, fibrinolysis, inflammation, and activation of the complement system, enzymatic processing of its C-terminal domain may convert prochemerin to chemerin, which has a much higher biological activity, such as chemerin-156 and chemerin-157 [20, 21]. However, different locations of extracellular C-terminal produce chemerin with different biological activities, such as chemerin-158, chemerin-155, chemerin-154, and chemerin-152, which show either low or unknown bioactivity [22, 23]. Intriguingly, the exact way in which prochemerin is cleaved involves the participation of multiple proteases, such as elastase and cathepsin G [24] and cathepsins L and K [25]. Besides the protease in the form of biological activity chemerin, many other proteases have been proved to be able to inactivate the biological activity of chemerin or process the prochemerin into a short inactive form of chemerin, thus competing with the activated protease for the prochemerin. In particular, chemerin-157 and chemerin-156 were hydrolyzed, by mast cell chymase, into a biologically inactive form, chemerin-154. Similarly, neutrophil protease 3 converted prochemerin into chemerin-155 [26] (see Figure 1). Too many C-terminal variations of chemerin and the relative activity of the isomers make it difficult to evaluate the biological activity of chemerin by immunological methods [27]. The diversity of chemerin isoforms reflects the multiplicity of its biological roles. The two opposite effects (pro- and anti-inflammatory) are related to the type of chemerin isomers [23, 24]. The visceral adipose tissue and liver are important sites for chemerin production [28, 29]. Low expression of chemerin is also found in the spleen, lymph nodes, upper lung, skeletal muscle, and *β* cells of the pancreas [30]. While plasma concentrations of prochemerin tend to be relatively high, those of bioactive chemerin isoforms are negligible under basic physiological conditions. Notably, the primary source of plasma prochemerin has not been identified [27].

Chemerin exerts its biological effects via receptor binding. Three receptors are currently known to bind chemerin: the chemokine-like receptor 1 (CMKLR1 or ChemR23), the G protein-coupled receptor 1 (GPR1), and the chemokine (C-C motif) receptor-like receptor 2 (CCRL2). De Henau et al. [31] revealed that chemerin had a similar affinity to CMKLR1 and GPR1 but a lower affinity to CCRL2. However, there are still practical problems in the study of chemerin receptors: these receptors display cell-specific expression profiles, which makes it difficult to compare the activation of receptors in the same tissue [32]. High levels of CMKLR1 transcription are detectable in white adipose tissue, macrophages, natural killer cells (NKs), immature dendritic cells (DCs), and leukocytes [33]. CMKLR1 mediates the chemotactic actions of chemerin, and it is currently the only receptor whose role as a mediator of chemerin's biological actions has been demonstrated convincingly [20]. It was also proved to be a G_i/o_ protein-coupled receptor that regulates angiogenesis and inflammation through mitogen-activated protein kinase (MAPK), extracellular signal-regulated kinase (ERK), and phosphatidylinositol 3-kinase (i.e., the PI3K/Akt pathways) [34]. GPR1 is primarily expressed not only in the central nervous system but also in skin cells, white adipocytes, and interstitial cells [35]. The existing studies showed that the GPR1- and CMKLR1-activated pathways appear to be responsible for chemerin's biological actions, and they both activate the ERK1/2-MAPK pathway [36], while the follow-up research about GPR1 still needs to be further promoted. CCRL2 is designated as an atypical, silent, or nonsignaled chemokine receptor. It concentrates chemerin on the cell surface, which facilitates the activation of CMKLR1 and GPR1 [37].

## 3. Chemerin and COPD-Related Inflammation

### 3.1. Intrapulmonary and Extrapulmonary Inflammation in COPD

Inflammation is the core pathology in COPD [38], which is closely related to the process of repair and remodeling of lung tissue. The destruction of the lung parenchyma caused by inflammation leads to emphysema and, ultimately, to irreversible airflow limitation [39]. Many cell types contribute to the inflammatory responses in COPD [40], including neutrophils, alveolar MFs, and CD8+ T lymphocytes. Neutrophils secrete elastase which may cause the destruction of the lung parenchyma and increase pulmonary secretion [41]. MFs recruit and activate neutrophils by releasing inflammatory mediators such as tumor necrosis factor (TNF) alpha, interleukin 8 (IL-8), and leukotriene B4 (LTB4) and maintain inflammation together with neutrophils [42]. Cytotoxic CD8+ T cells induce apoptosis of alveolar epithelial cells [43]. Moreover, the activation of Toll-like receptors (TLR) by these inflammatory cells enables the recognition of pathogenic microorganism-related molecular patterns and damages related molecular patterns, and the body's immune response to inflammation is activated [44]. Typically, neutrophils and MFs initiate an innate immune response [45, 46]. This process is accompanied by the release of oxygen free radicals and proteolytic enzymes, resulting in the production of a large number of mucins and inducing airway obstruction [47, 48]. In addition, the acquired immune response will be activated once the uncontrollable innate immune response occurs, thus amplifying COPD inflammation [49].

Recently, the extrapulmonary inflammatory response of COPD has been paid more attention, which can be manifested as the increase of circulating cytokines, chemokines, and acute-phase proteins or the abnormality of circulating cells [50]. “Overspill” of inflammatory factors has been proposed to explain this phenomenon [51]: inflammatory factors in the lungs spread to the entire body by means of the circulatory system. However, it should be noted that the accuracy of this hypothesis needs to be further proved. Fortunately, it is certain that many types of extrapulmonary injury of COPD are related to systemic inflammation, for example, skeletal muscle atrophy, respiratory muscle dysfunction [52], and osteoporosis [5]. In terms of skeletal muscle dysfunction in COPD, one research revealed the negative correlation between skeletal muscle strength and markers of systemic inflammation during acute exacerbations in COPD patients [53]. Also, systemic inflammation may evoke an imbalance of the synthesis and degradation of muscle protein [54], thereby promoting further skeletal muscle dysfunction. Proinflammatory mediators (such as TNF-*α*) enhance the activity of the ubiquitin-proteasome system (UPS) by activating nuclear factor-kappa B (NF-*κ*B), which facilitates the occurrence of muscle atrophy [55]. Thus, improved control of inflammation could therefore reduce additional, long-term pathological effects of COPD to the body.

### 3.2. Dual Effects of Chemerin on Inflammation

As confirmed for many inflammatory diseases [56–58], the present understanding is that chemerin has a dual effect on inflammation. Importantly, it regulates the recruitment of leukocytes, especially DCs, MFs, and NKs, towards inflammatory sites. Interestingly, it may also inhibit the synthesis of proinflammatory mediators, thus serving anti-inflammatory roles. These two contrary effects of chemerin have distinct prominence during different stages of the inflammatory response and are also related to the internal and external environment of the organism, such as the disease state of the organism and the types of the intervention [14, 28].

**Figure 2:**
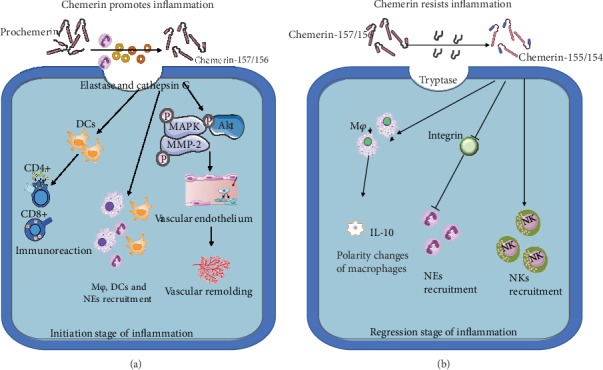
Roles of chemerin in chronic inflammation. (a) Stimulation of chronic inflammation by chemerin. The presence of neutrophils induces the formation of chemerin homologues, which recruit the local aggregation of additional inflammatory cells. This affects vascular remodeling by acting on vascular endothelial cells. These chemerin isomers further recruited antigen-presenting cells and neutrophils. Antigen-presenting cells initiate further immune responses. (b) Inhibition of inflammation by chemerin. Several serine proteases (tryptase) produce chemerin homologues with anti-inflammatory activities. These molecules change the polarity of macrophages and induce their anti-inflammatory effects. Meanwhile, they inhibit the aggregation of inflammatory cells such as neutrophils and activate NK cells to promote the dissipation of inflammation. Abbreviation: NEs: neutrophils; M*φ*: macrophages; NKs: natural killer cells.

Elevated levels of chemerin are often found in chronic inflammatory diseases [59]. For example, immunoreactive chemerin has been found in fibroblasts, mast cells, and endothelial cells during early inflammation-related pathological changes [33, 60]. During the early stages of inflammation, chemerin promotes the development of inflammation. The earliest appearance of polymorphonuclear cells releases elastase and cathepsin G, which induces chemerin to produce biologically active isomers of chemerin (chemerin-157 or chemerin-156) at inflammatory sites [61]. Biologically active chemerin firstly causes vascular endothelial dysfunction and construction of new blood vessels. Vascular response is the earliest manifestation of inflammation, and the destruction of the vascular basement membrane enhances the degree of inflammation [62]. Under the regulation of proinflammatory factors, chemerin in endothelial cells guides the formation of new blood vessels through a variety of pathways, such as MAPK, Akt, and activation of endothelial gelatinases (MMP-2/-9) [12]. Meanwhile, these isomers of chemerin also promote additional recruitment of antigen-presenting cells (APC) and probably also neutrophils, thus exacerbating inflammation. In addition, APC, such as DCs and MFs, are recruited in response to activation by chemerin connecting innate and adaptive immunity at the beginning of the immune response, and the basis of this phenomenon is that CMKLR1 is expressed on DCs although it is associated with the maturation state of DCs. Chemerin also promotes the migration of DCs from the circulation to reactive lymph nodes [13, 63]. Fully mature dendritic cells acquire antigen-presenting cell phenotype and upregulate major histocompatibility complex class I and II and T cell costimulatory molecules, which drive CD4+ and CD8+ T cell activation although lose CMKLR1 expression on their surface [61] (see Figure 2(a)). Thus, chemerin plays a central role not only in regulating the recruitment of DCs in inflammatory sites but also in triggering innate immunity [64].

Notably, a large number of studies have reported the anti-inflammatory effects of the chemerin-CMKLR1 axis. The earliest report, which involved a peritonitis model, showed that chemerin inhibits the aggregation of neutrophils and monocytes and decreases the expression of proinflammatory cytokines [65]. Chemerin's anti-inflammatory effects are especially prominent during the regression period of inflammation. Chemerin, formed by a serine protease reaction, may be converted by other serine or cysteine proteases to homologous peptides such as chemerin-155 or chemerin-154, which are inactive, nonchemotactic, or anti-inflammatory. For example, tryptase, a serine protease in mast cells, converts prochemerin to chemerin-158 and chemerin-155, while mast cell chymase converts active chemerin-157 and chemerin-156 to inactive chemerin-154 [66]. Neutrophil-derived serine protease 3 is also considered to have a similar function [26]. In addition, various isomers of chemerin have different effects on the polarization of MFs to promote or resist inflammation [67, 68]. It means that MFs with anti-inflammatory effects are more activated by some chemerin isomers [67]. Chemerin-induced expression of anti-inflammatory cytokines such as IL-10 in MFs has also been reported [69], although this is inconsistent with the study that stimulating MFs with active chemicals increased the expression of proinflammatory cytokines [67]. The result of this contradiction may be caused by the participation of different chemerin isomers. Moreover, some types of chemerin homologues attenuate the direct recruitment of neutrophils by inhibiting integrin activation and promote the clearance of neutrophils or apoptotic cells from mucosal surfaces, thereby facilitating the dissipation of acute inflammation [70]. Finally, chemerin also regulates immune responses by affecting NK cells [33], thus affecting inflammation. NK cells play an important role in the early stage of innate immune response and in the process of solving inflammation by restricting adaptive immune response. The expression of CMKLR1 in NK cells enabled it to migrate to the site where inflammation dissipated. The role of chemerin in DCs and NK cells in pathological peripheral tissues revealed by relevant studies further confirms this view: chemerin moderates adaptive immune responses by transporting NK cells to injured sites [71]. Overall, chemerin plays a significant role in the resolution of inflammation (see Figure 2(b)), but it is worth noting that chemerin's anti-inflammatory effects critically depend on the rate of synthesis of selected chemerin homologous peptides, which is determined by the activity of serine and cysteine proteases [64].

### 3.3. Effects of Chemerin on COPD-Related Inflammation

Clinical studies have demonstrated that chemerin participates in pathological processes during acute exacerbation of COPD. Levels of chemerin in peripheral blood of patients with acute exacerbation were found to be significantly higher than those of healthy controls [72]. With recovery from the exacerbation, the expression level of chemerin decreases, and the expression of chemerin in serum is positively correlated with the leukocyte count and C-reactive protein levels in peripheral blood [72]. Boyuk et al. [73] also found increased plasma levels of chemerin in COPD patients and no significant difference in the expression of chemerin in patients at different stages of pulmonary functioning. Analysis of smoking history in the COPD group revealed no difference in chemerin expression between patients who had quit smoking and patients who had not. A retrospective study in COPD patients [74] showed that treatment with salmeterol/fluticasone propionate combined with aerosol inhalation significantly reduced plasma levels of IL-8 and C-reactive protein in COPD patients and that plasma chemerin levels were lower after treatment than before. Notably, the existing research did not identify the specific isoforms of chemerin, which also becomes the potential direction of future research.

Animal studies confirmed both anti-inflammatory and proinflammatory effects in pulmonary inflammatory processes. In a model of pulmonary inflammation established by cigarette smoke exposure, the expression levels of prochemerin in airway epithelial cells were significantly reduced, but the level of chemerin secreted into the airway lumen was increased, indicating the proinflammatory effect of chemerin. Meanwhile, the lung parenchyma of mice was infiltrated by inflammatory neutrophils, as well as activated DCs and CD4+ T cells [75]. Still in the same experiment, the expression of chemokines in CMKLR1^(-/-)^ mice was significantly lower than that in wild-type (WT) mice. Also, 14 days after smoking exposure, T cell aggregation in BALF of CMKLR1^(-/-)^ mice was significantly delayed, while 14 days after smoking exposure in WT mice, the presence of inflammatory cells in the airways and lymph nodes persisted [75]. Intriguingly, the anti-inflammatory effect of chemerin was found in mice with viral pneumonia [76]: CMKLR1^(-/-)^ mice displayed higher mortality/morbidity, alteration of lung function, delayed viral clearance, and increased neutrophilic infiltration; meanwhile, lower recruitment of plasmacytoid dendritic cells and a reduction in type I interferon production were also observed in these mice. In addition, an opposing role for CMKLR1 signaling depending on the context of inflammation was found [77]: the CMKLR1 axis showed proinflammatory properties in a model of diesel exhaust particle- (DEP-) induced acute lung inflammation, in contrast to anti-inflammatory effects in a model of DEP-enhanced allergic airway inflammation. In summary, existing studies primarily confirm chemerin's inflammation-related effect in COPD; however, the role of the isomers in COPD still needs to be further explored.

## 4. Chemerin Involved in Glucose and Lipid Metabolism in COPD

### 4.1. Abnormal Glucose and Lipid Metabolism in COPD

As mentioned earlier, COPD is known to be associated with abnormalities in metabolic phenotype [78], including weight loss and skeletal muscle atrophy [79, 80]. However, the current global epidemic of obesity changes the nature of the nutritional abnormalities observed in COPD: larger proportions of COPD patients are currently overweight or obese than underweight, and the combination of COPD and obesity is increasing [81, 82]. Overweight and obesity are associated with prolonged survival compared with those with lower body mass index (BMI) [83], especially in severe disease in COPD [84, 85], which was called the “obesity paradox.” Nevertheless, the harmful long-term effects of obesity-related conditions such as low-grade systemic inflammation [86] and insulin resistance [87] may result in increased cardiovascular and all-cause mortality. The factors that cause this phenomenon may be complex [88], and the underlying pathophysiology remains unclear. Hence, monitoring of metabolic phenotype in COPD patients could be expected to enhance the survival rate of COPD, and this section provides a brief summary of existing research.

Hyperglycemia and glucose intolerance are frequent findings in nondiabetic patients with chronic disease. A similar situation has been shown in patients with COPD, manifested by the marked acceleration of endogenous glucose flux and clearance, in particular via increases of glycolysis and glucose oxidation [89]. Meanwhile, abnormal glucose metabolism was also detected in the respiratory muscles of COPD patients: the rate of glucose metabolism in the diaphragm was significantly higher than that in other peripheral muscles [90]; Ferguson et al. believed that it is correlated with the mechanical loads confronted [91]. The carbohydrate metabolic abnormality may be related to many factors. First, insulin resistance seems to be at the basis of this metabolic disturbance [92]. Patients with chronic diseases are more likely to develop peripheral insulin resistance than healthy people, just as COPD patients [93]. Bolton et al. [93] assessed the association between systemic inflammation and insulin resistance in nonhypoxaemic patients with COPD. They demonstrated greater insulin resistance in nonhypoxaemic patients with COPD compared with healthy subjects, which was related to systemic inflammation. Second, hypoxia plays an important role in the formation of abnormal glucose metabolism in patients with poor pulmonary function. As pulmonary function deteriorates and disease progresses, the risk of alveolar hypoxia and consequent hypoxemia increases [94]. Intermittent hypoxia commonly occurs during low-intensity daily activities (such as eating and walking), although oxygen levels may be near normal levels during rest [95]. Hypoxia stimulates glucose production, and tissues are more likely to ingest glucose at lower blood glucose levels [96]. Finally, medications for COPD patients also affect glucose metabolism: Wu [97] concluded that long-term glucocorticoid therapy also impaired fasting blood glucose and glucose tolerance in COPD patients, thus facilitating abnormal glucose metabolism in COPD patients.

Obese COPD patients are also commonly associated with abnormal lipid metabolism [81]. The data from 11 868 adults in the 2012 South Carolina Behavioral Risk Factor Surveillance System survey showed that persons who were at extreme ends of BMI (underweight, obese, or morbidly obese) had a higher prevalence of COPD (13.1, 8.5, and 15.4%, respectively) than persons at a normal weight (6.7%) [98]. As for the factors that contributed to abnormal lipid metabolism in COPD, hypoxia may be one of them. Since adipose tissue blood flow is reduced in COPD humans, adipose tissue hypoxia may induce increased secretion of inflammatory factors, acute-phase proteins, and angiogenic factors by adipose tissue in order to increase blood flow and vascularisation, resulting in adipose tissue as a source of inflammation in COPD [99]. Second, intermittent hypoxia in COPD may mediate insulin resistance through adipose tissue inflammation [100]. Potential mechanistic links between systemic inflammation, insulin resistance, and hypoxic environment in COPD patients are the potential inducements of fat metabolism disorder. Finally, adipokines [101], in particular, adiponectin and leptin, playing a significant role in the regulation of inflammation and maintenance of lipid metabolism balance [101], were thought to contribute to abnormal lipid metabolism [102]. Increased levels of inflammatory factors in COPD promoted the secretion of a large number of adipokines in the body, resulting in disordered body fat metabolism [103]. In other studies, obesity has been shown to have a negative effect on functional outcomes such as 6 min walk scores in patients with COPD [104]. Thus, it is speculated that, compared with the combined increase of fat and nonfat body weight, improving muscle mass is a better way to improve the survival rate of COPD.

### 4.2. Chemerin Regulates Glucose and Lipid Metabolism in COPD

**Figure 3:**
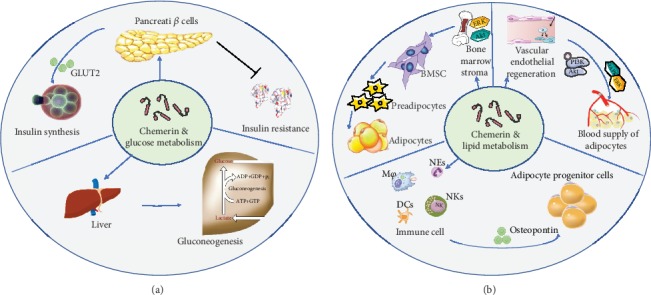
Summary of ways in which chemerin affects glucose and lipid metabolism. (a) Roles of chemerin in glucose metabolism. Chemerin is expressed in pancreatic beta cells and regulates glucose metabolism via effects on insulin synthesis and insulin resistance. Additionally, the effect of chemerin on gluconeogenesis is one of its mechanisms. (b) Roles of chemerin in lipid metabolism and adipogenesis. Firstly, chemerin stimulates the conversion of BMSC to adipocytes. Secondly, chemerin indirectly stimulates lipid metabolism by enhancing the blood supply of adipose tissue in a chronic low-level inflammatory environment. Abbreviation: NEs: neutrophils; M*φ*: macrophages; NKs: natural killer cells; BMSC: bone marrow mesenchymal stem cells; GLUT2: glucose transporter 2.

Adipokines, including chemerin, have been shown to regulate glucose homeostasis and lipid storage [28, 102, 105]. Intriguingly, both inhibitory [106] and stimulatory [107] effects of chemerin on glucose homeostasis were found, and the mechanism is still unclear. However, this regulatory approach of chemerin has several points in common. First, chemerin regulates insulin secretion and insulin sensitivity. Further studies showed that glucose-induced insulin release in CMKLR1 knockout mice is reduced [108]. Mechanistically, the deletion of chemerin downregulates the expression of transcription factor MAFA and its downstream target gene product, glucose transporter 2 (GLUT2) [109]. In addition, GPR1 shows effects similar to those of CMKLR1 [105]. Second, chemerin affects insulin resistance. An animal experiment found that chemerin treatment aggravates glucose intolerance in obese and diabetic mice [110]. *In vitro* study demonstrated that chemerin induces insulin resistance in human skeletal muscle cells at the level of insulin receptor substrate-1, Akt and glycogen synthase kinase 3 phosphorylation, and glucose uptake [111]. However, chemerin knockout mice show impaired insulin sensitivity in the adipose tissue and liver [59] and decreased glucose uptake in the adipose tissue and skeletal muscle [109]. Loss of CMKLR1 functioning increases insulin resistance in mice fed a high-fat diet [112]. Ernst et al. believed that the different concentrations, treatment durations, and conditions might have contributed to the discrepant results [28]. Finally, chemerin acts on gluconeogenesis, and chemerin knockout mice show upregulated key regulatory factors involved in gluconeogenesis, including glucose-6-phosphatase and phosphoenolpyruvate carboxylase [70], with an inhibitory effect of insulin on hepatic glucose production (see Figure 3(a)).

Chemerin is highly expressed in white but lowly in brown adipocytes. Based on the correlation between brown fat and heat production, it has been speculated that chemerin is mainly related to regulating adipogenesis rather than heat production [16]. During the differentiation of human preadipocytes into adipocytes, the chemerin-CMKLR1 axis activated [113], and the process depends on Akt-mTOR and ERK signaling cascades [114]. Inhibition of chemerin also prevents the transformation of myoblasts from myogenesis to adipogenesis [115]. Activation of chemerin prompts bone marrow mesenchymal stem cells (BMSC) to convert into adipocytes rather than into osteoblasts [29, 116]. Furthermore, during inactivation of the chemerin signaling by gene regulation or antibody neutralization, the pattern of BMSC-related activities shifted from adipocyte differentiation and proliferation to osteoblast differentiation [117, 118]. In contrast, other studies indicate that CMKLR1 knockout mice show no inhibition of adipogenesis, a result that was confirmed *in vitro* [119]. These observations might be related to the complex organization of adipose differentiation and metabolism. In addition, the expression of chemerin in the vascular endothelium may affect fat metabolism by changing the blood supply to adipose tissue [120]. It promotes the proliferation, differentiation, capillary formation, and migration of endothelial cells through PI3K-Akt and MAPK-ERK signaling [121]. Therefore, the activation of chemerin signaling also enriches the supply of lipid vessels and ultimately promotes the growth of lipids. Finally, chemerin also involves in adipogenesis as a chemokine [122], since the current study indicated that the increased adiposity is thought to be associated with chronic low-grade systemic inflammation [123]. Another study confirmed that MFs promote the recruitment, proliferation, and differentiation of adipocyte progenitor cells by secreting osteopontin [124]. Local and systemic inflammation caused by adipocyte-immune cell crosstalk significantly leads to insulin resistance and abnormal lipid metabolism, which is evident in COPD patients. Hence, chemerin also regulates lipid metabolism by acting via inflammatory processes, especially on immune cells (see Figure 3(b)).

Currently, there are few direct studies on changed chemerin levels and their effect in COPD glucose and lipid metabolism. However, judging by the significant effects of glucose and lipid metabolism on the course of COPD as well as by chemerin's effect on glucose and lipid metabolism, chemerin potentially represents a vital target for therapeutic intervention in COPD patients. A related study [125] showed that the level of plasma chemerin in COPD patients was higher and negatively correlated with blood levels of various lipids: total cholesterol (TC), triglyceride (TG), and high-density lipoprotein (HDL), whereas a positive correlation with low-density lipoprotein (LDL). The increased plasma levels of chemerin in COPD patients could reflect the level of their lipid metabolism, which guides the intervention of metabolic dysfunction. It was also found that circulating levels of both chemerin and lipids, including TC, TG, HDL, and LDL, are related to the six-month readmission and mortality rates of COPD patients. This suggests that chemerin might be used as an index for health status assessment and prognosis [125]. Another study showed that salmeterol/fluticasone propionate combined with pursed lip breathing effectively reduced plasma chemerin and lipid levels in COPD patients [74]. In sum, although existing studies indicate that chemerin is involved in abnormal glucose and lipid metabolism in COPD, more detailed studies of the underlying mechanisms are needed.

## 5. Exercise-Induced Changes in Chemerin in the Rehabilitation of COPD

### 5.1. Inflammation and Glucose-Lipid Metabolism

In COPD, different pathologies coexist and interact, such as inflammation and metabolic perturbations. Over the past few years, the role of chronic inflammation in the pathogenesis of glucose metabolism has received increasing attention, with an emphasis on insulin resistance [93]. TNF-*α* induces insulin resistance by promoting lipolysis of adipocytes and increasing serine/threonine phosphorylation of insulin receptor substrate-1 (IRS-1) [126]. It also has been reported to increase glucose uptake in visceral and subcutaneous adipocytes by activating the adenosine monophosphate-activated protein kinase (AMPK) pathway, thereby activating c-Jun N-terminal kinase 1/2 and triggering insulin resistance in visceral adipocytes [127]. Other cytokines have also been shown to link inflammation to insulin resistance, such as IL-1*β*, IL-6, and leptin [128–130]. The application of salicylic acid as an anti-inflammatory agent to improve glucose metabolism in type 2 diabetes mellitus also illustrates the close interrelationship between inflammation and glucose homeostasis [131, 132]. Hence, it could be concluded that there is a crosstalk between signaling pathways that are active in inflammation and those activated by the insulin receptor.

The interactive relationship was found between inflammation and lipid metabolism. On the one hand, abnormal lipid metabolism may induce inflammation. Firstly, the abnormal modification and localization of lipoprotein regulate inflammatory response [133, 134]. In the absence of other inflammatory stimuli, excessive free cholesterol in the cell membrane or connotation can activate the p38 MAPK signaling pathway through TLR3 or TLR4 pathway to induce the inflammatory response [133]. As important tools for cholesterol to enter and exit cells, the imbalance between HDL and LDL activates many pathways [134] (including unfolded protein response, p38 MAPK, and NF-*κ*B), inhibits the expression of anti-inflammatory factors, and intensifies inflammatory response [135]. Secondly, studies showed that abnormal lipid metabolism can affect inflammation through macrophage polarization [136, 137]. According to the polarization type, macrophages can be divided into M1 and M2. The former mainly produces proinflammatory cytokines, while the latter produces anti-inflammatory cytokines. These two polar macrophages are present simultaneously in atherosclerotic lesions [136], suggesting that abnormal lipid metabolism may lead to the transformation of M2 to M1 [137]. Finally, abnormal lipid metabolism can also induce inflammation through nucleotide-binding oligomerization domain-like receptor family pyrin domain-containing 3, which is confirmed in atherosclerosis [138]. On the other hand, inflammation aggravates abnormal lipid metabolism. Inflammation promotes the uptake and accumulation of lipids by cells: inflammatory factors IL-1*β* and TNF-*α* destroy the negative feedback regulation mechanism of LDL and make LDL receptor continuously express [139], resulting in excessive accumulation of lipids in cells. Moreover, HDL hydrolysis is accelerated in an inflammatory state, and the activity of cholesteryl ester transfer protein and lecithin cholesterol acyltransferase decreases [140], which reduces the outflow of cholesterol to cells. In this process, the pathological modification of HDL was found [141], which means that dysfunctional HDL increases in the inflammatory response. Given this close correlation between inflammation and glucose and lipid metabolism, improving the control of inflammation and the regulation of metabolism is likely to benefit the treatment and rehabilitation of COPD patients.

### 5.2. The Regulating Effect of Exercise on Chemerin

Since their initial discovery, the various chemerin-mediated effects on inflammation and metabolic disorders have attracted an ever-increasing attention from researchers [16, 27]. With the deepening of research, some effective interventions have been found to be able to effectively change the expression of chemerin. Importantly, appropriate exercise training has been proven to be an effective way to regulate the expression of chemerin and reduce the risk of metabolic diseases [142]. This may be due to the fact that exercise is one of the few nondrug interventions that affect adipose tissue physiology. We screened the clinical studies of the exercise intervention on the changes of chemerin from 2010 to 2019 (published in English) and summarized the representative studies (see Table 1). The existing research data showed that long-term regular exercise indeed caused the change of chemerin *in vivo*. Most of the related researches focus on obesity and type 2 diabetes, which may be related to the important role of exercise in the management of chronic metabolic diseases. As for the exercise prescription, the types of exercise involved aerobic and strength training, and both of them caused the change of chemerin. Kim et al. [143] confirmed that only combined exercise (aerobic combined with resistance training) had a negative impact on chemerin expression. In addition, the duration of exercise intervention varied in these studies, and even a single exercise had an impact on the level of chemerin. Notably, the exercise-induced changes in chemerin are closely related to body fat and insulin resistance. For example, after six months of exercise intervention, the decrease of chemerin in obese patients was closely related to the changes in waist circumference, fat mass, LDL cholesterol, and insulin resistance index of homeostasis model assessment (HOMA-IR) [144]. Moreover, Nordic walking effectively downregulated plasma levels of chemerin, TC, and LDC in obese men, while alleviating several characteristic parameters of metabolic syndrome [145]. The changes of chemerin were also associated with TC and TG concentrations and first-phase and total glucose-stimulated insulin secretion (GSIS) after exercise [146]. Related studies listed chemerin as the final index and also indicated that the effect of exercise on chemerin is one of the potential mechanisms of exercise intervening obesity and type 2 diabetes [143, 147].

However, the precise identification of specific mechanisms by which exercise regulates chemerin expression requires additional research. *In vivo*, chemerin is expressed in many tissues, although the expression levels vary (described earlier). The existing clinical research shows that the effect of exercise on chemerin is related to body fat [144] and insulin resistance [148], which suggests that the upstream signal of exercise regulating chemerin may be related to fat and insulin. Nevertheless, the hypothesis is still open to further discussion. Moreover, exercise is not a single-target intervention [149, 150], which implies that the specific pathways by which exercise regulates chemerin production are likely to be diverse. PPAR*γ* is considered an upstream regulator of chemerin activity [116]. After PPAR*γ* is activated, it forms a heterodimer with retinoid X receptor (RXR) and binds with PPAR response element (PPRE), thereby regulating the expression of metabolic-related proteins or enzymes including chemerin [151]. Lin et al. further confirmed this point in animal studies: after exercise, the level of chemerin/CMKLR1 in the serum, liver, and gastrocnemius of obese and type 2 diabetic mice decreased significantly, which could be reversed by PPAR*γ* antagonist GW9662 and further strengthened by PPAR*γ* agonist pioglitazones [116]. However, further study of the effects of exercise on the upstream signal pathway of PPAR*γ*, such as the Wnt signaling pathway [152], should help to reveal the mechanisms by which exercise affects chemerin in more detail. Notably, current difficulties in measuring which of the different forms of chemerin are regulated by exercise remain a major problem, especially because different isoforms of chemerin not only are produced under different circumstances but also have different—sometimes even opposite—physiological roles [28]. This mechanism may also involve the change of different isoforms from prochemerin to chemerin.

**Figure 4:**
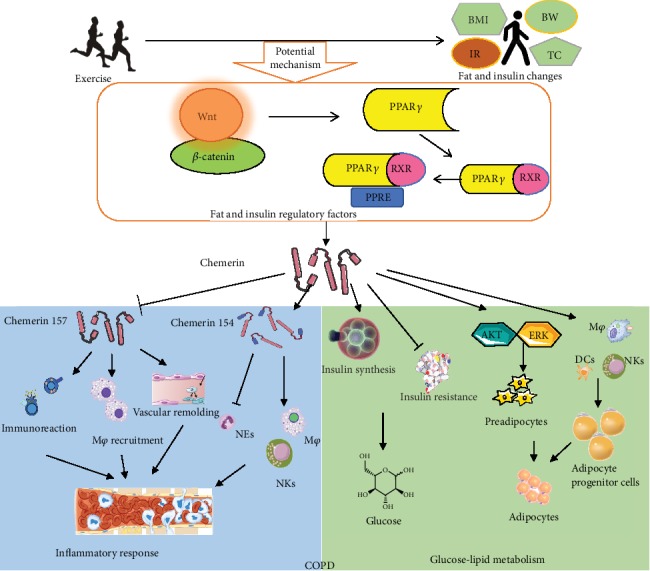
Exercise-induced chemerin signaling may mediate pulmonary rehabilitation. The beneficial effects of exercise on inflammation, glucose metabolism, and lipid metabolism may be achieved by altered expression of chemerin. Different chemerin isoforms are likely to exhibit either anti-inflammatory or proinflammatory roles, while exercise may selectively inhibit the proinflammatory effects and promote the expression of anti-inflammatory forms of chemerin. Exercise may also improve glucose metabolism via interactions of the chemerin and insulin signaling pathways. Finally, exercise improves lipid metabolism by effects on adipocyte differentiation and immune reaction. The regulation of exercise on chemerin may be related to the effect of exercise on fat and insulin. Wnt and PPAR*γ* may be involved in the regulation mechanism of exercise on chemerin. Abbreviation: BMI: body mass index; BW: body weight; TC: total cholesterol; IR: insulin resistance; PPAR*γ*: peroxisome proliferator-activated receptor gamma; RXR: retinoid X receptor; NEs: neutrophils; M*φ*: macrophages; NKs: natural killer cells; DCs: dendritic cells; COPD: chronic obstructive pulmonary disease.

As a common approach to lung rehabilitation, exercise plays a crucial role in the prevention and treatment of COPD and related functional disorders. Exercise improves the lung function of COPD patients [153] and is considered to be the only nondrug intervention that can effectively reverse respiratory muscle dysfunction in patients [154]. It does so by changing the structure of the respiratory muscle [155, 156]. Exercise may alleviate skeletal muscle dysfunction and atrophy in COPD patients [157, 158]. Besides, it has a pronounced effect on the metabolism of glucose [89, 159] and lipids [160] in COPD patients. The mechanism by which exercise improves COPD dysfunction may be related to its effects on inflammation: exercise regulates chronic inflammation [161–163]. Davidson et al. [164] found that exercise inhibits the level of inflammatory factors in COPD sputum, which indicates an anti-inflammatory effect. Considering the physiological role of chemerin and the stimulating effect of exercise on chemerin levels in COPD patients, beneficial effects of exercise on COPD may be mediated in part via chemerin-dependent mechanisms (see Figure 4). However, at this moment, direct evidence in support of this hypothesis is still lacking and awaits confirmation in future studies. In conclusion, exercise may beneficially affect glucose metabolism, lipid metabolism, and inflammation in COPD patients by changing the expression of chemerin, which exhibits pulmonary rehabilitation effects of exercise. This makes chemerin a potential mediator of—and a therapeutic target for—lung rehabilitation.

## 6. Conclusions and Prospects

Inflammation and disordered glucose and lipid metabolism are essential effects of COPD. Given the interaction between inflammation and glucose and lipid metabolism, simultaneous control of inflammation and metabolism is an effective way of reducing the negative impact of COPD on patients' health. The chemerin/CMKLR1 axis contributes to both inflammation and metabolism in various diseases, including COPD. However, more direct evidence and details of chemerin involved in COPD are still worthy of further discussion. Of potentially great clinical relevance, exercise training has a pronounced, inhibiting effect on chemerin, and this may represent a key mechanism in exercise-induced, pulmonary rehabilitation. Future studies should further elucidate the mechanisms of exercise regulation of chemerin, while providing guidance for chemerin-based approaches in pulmonary rehabilitation.

## Figures and Tables

**Table 1:** Summary of studies on the regulation of chemerin by exercise.

Study ID	Design	Sample size	Participants	Intervention	Chemerin level	Other findings related to chemerin changes
Malin et al. [146]	Prospective clinical trial	Total: 30	Older obesity adults	Components: treadmill walking and cycle ergometer exercise Frequency and duration: 5 days/wk; 60 minutes/day; 12 wks Intensity: 85% of MHR	Decreased chemerin compared to the baseline (78.1 ± 5.8 vs. 87.1 ± 6.0 ng/ml; *P* = 0.02)	BW (*r* = 0.39, *P* = 0.03); total body fat (*r* = 0.42, *P* = 0.02); VF (*r* = 0.50, *P* = 0.009); TC (*r* = 0.38, *P* = 0.04); triglyceride concentrations (*r* = 0.36, *P* = 0.05); first-phase and total GSIS (*r* = 0.39, *P* = 0.03 and *r* = 0.42, *P* = 0.02)

Aghapour and Farzanegi [165]	Quasiexperimental research with a pre-post test	Total: 20 EG (*n* = 10); CG (*n* = 10)	Hypertensive postmenopausal women	EG Components: warm-up, main training exercises, and cool-down exercises Frequency and duration: 3 times a wk; 60 minutes/day; 6 wks Intensity: 50% MHR and 5% was added every 2 wks CG did not participate in any training program	Decreased chemerin level compared to the baseline (250.66 ± 31.22 vs. 337.48 ± 35.56 pg/ml; *P* ≤ 0.001)	—

Faramarzi et al. [148]	Clinical controlled trial	Total: 35 EG (*n* = 19); CG (*n* = 16)	Overweight women	EG Components: warm-up, rhythmic AE, core stability training, cooling processes Frequency and duration: 3 times a wk; 80 minutes/day; 12 wks Intensity: 30 at 55% of heart rate reserve and progressed to 80% of heart rate CG did not participate in any training program	A significant difference in serum chemerin level in EG vs. CG (408.75 ± 218.4 vs. 336.56 ± 189.43 mg/dl; *P* = 0.008)	BMI, WC, body fat, insulin, and IR also show the significant difference in the comparison (EG vs. CG)

Stefanov et al. [144]	Clinical controlled trial	Total: 56 EG (*n* = 28); CG (*n* = 28)	Overweight or obese individuals	EG Components: AE, RE, and flexibility exercises Frequency and duration: 2 times a wk, 30-35 min/day (1-10 wk); 3 times a wk, 45-60 min/day (11-20 wk); 4 times a wk, 45-60 min/day (21-24 wk) Intensity: AE at 50-60% of the MHR; RE for the major muscle groups in two sets of 8-14 repetitions (1-10 wk); AE at 60-75% of MHR; RE in three sets of 8-14 repetitions (11-24 wk) CG: usual care and education	Chemerin decreased significantly from baseline within the exercise group (post-pre) (−13.8 ± 13.2 ng/ml, *P* = 0.002).	WC (r = 0.34, *P* = 0.01); fat mass (*r* = 0.40, *P* < 0.01); fat-free mass (*r* = −0.37, *P* < 0.01); HOMA-IR (*r* = 0.38, *P* < 0.01); LDL cholesterol (*r* = 0.28, *P* < 0.01)

Neuparth et al. [166]	Cross-sectional and retrospective study	Total: 83 Active group: 30 Sedentary group: 51	Type 2 diabetes mellitus patients	Active group Components: walking exercise Frequency and duration: three times a wk, at least 30 min/day, 1 year Intensity: moderate	Chemerin level in active group vs. sedentary group (134 (102-181) vs. 181 (156-199) ng/ml, *P* = 0.001)	BMI, body weight, and oxidized LDL also show a significant difference in the comparison (active group vs. sedentary group)

Venojarvi et al. [145]	Randomized controlled trial	Total: 144 Nordic walking group (*n* = 48); resistance training group (*n* = 49); CG (*n* = 47)	Overweight and obese men	Nordic walking group Components: warm-up, AE, cool-down Frequency and duration: 3 times a wk, 60 min/day, Intensity: 55% to 75% of MHR Resistance training group Components: warm-up, RE, cool-down Frequency and duration: 3 times a wk, 60 min/day, 12 wks Intensity: 50%-85% from exercise-specific maximal strength (5RM) CG had no supervised exercise	Both exercise groups downregulated serum chemerin (post-pre) compared to CG (-1.6 (2.7), -1.1 (3.8), and 8.1 (2.6) *μ*g/ml)	TC, fatty liver index, LDL cholesterol, BW, leptin, and fat percentage decreased in Nordic walking group (vs. CG)

Kim et al. [167]	Randomized controlled trial	Total: 35 EG (*n* = 18); CG (*n* = 17)	Overweight or obese subjects with type 2 diabetes	EG Components: AE; RE (warm-up, core exercises, resistance training, cool-down) Frequency and duration: 2-3 times a wk, 30-40 min/day (AE); 3 times a wk, 50 min/day (RE); 12 wks Intensity: 50-70% of the MHR (AE); 3 sets of 20 repetitions at 50% 10RM (RE) CG: usual care and education	A significant decrease (post-pre) was showed in the comparison between the exercise vs. control groups (−6.4 ± 28.5 vs. 14.5 ± 21.5 ng/ml, *P* = 0.026)	ISI (*β* = −0.39, *P* = 0.01); lipocalin-2 (*β* = 0.33, *P* = 0.008); FPG (*β* = 0.32, *P* = 0.029); TC (*β* = 0.26, *P* = 0.038)

Saremi et al. [142]	Randomized controlled trial	Total: 21 EG (*n* = 11); CG (*n* = 10)	Overweight and obese subjects	Exercise group Components: warm-up, treadmill walking-running, relaxation exercises Frequency and duration: 5 days a wk, 50-60 min/day, 12 wks Intensity: 15-20 min at 60-65% of HRmax (wk 1); 25-30 min at 60-70% of MHR (wk 2-3); 35-40 min at 75-80% of MHR (wk 4-7); 45-50 min at 80–85% of MHR (wk 8-12) CG: usual lifestyle	A significant decrease was showed in the comparison between two groups (*P* = 0.02)	VF (*r* = 0.65, *P* = 0.04), subcutaneous fat (*r* = 0.63, *P* = 0.05), HOMA-IR (*r* = 0.67, *P* = 0.04), glucose (*r* = 0.65, *P* = 0.05), WC (*r* = 0.70, *P* = 0.03), and VO_2_ peak (*r* = −0.68, *P* = 0.04)

Kim et al. [143]	Randomized controlled trial	Total: 47 CG (*n* = 15); RE group (*n* = 16); combined exercise group (*n* = 16)	Healthy elderly participants	RE group Components: leg extension, pull weight, chair pull, cool down Frequency and duration: 3 days a wk, 70 min/day, 6 wks Intensity: Borg scale 6 (1-2 wks); Borg scale 7 (3-4 wks); Borg scale 8 (5-6 wks) Combined exercise group Components: leg extension, pull weight, chair pull, sky-walk, cross-country, cool down Frequency and duration: 3 days a wk, 90 min/day, 6 wks Intensity: Borg scale 6 (1-2 wks); Borg scale 7 (3-4 wks); Borg scale 8 (5-6 wks) CG: usual care	A significant reduction in chemerin level (post-pre) in combined exercise group vs. CG (−14.51 ± 27.75 vs. 11.04 ± 15.17 ng/ml)	Significant reductions in fasting insulin and HOMA-IR were observed in the combined exercise group

Supriya et al. [168]	Randomized controlled trial	Total: 97 CG (*n* = 45); yoga groups (*n* = 52)	Individuals with metabolic syndrome and high-normal blood pressure	Yoga group Components: warm-up, hatha yoga practice, cool-down Frequency and duration: once a wk, 60 min/once, 1 year Intensity: — CG: contacted monthly to monitor their health status	Significant reduction in chemerin level was observed in the yoga group vs. the control group	Significant reduction in WC was observed in yoga vs. CG

Data are presented as mean ± SD, means (SE), or median values (interquartile ranges). EG = exercise group; CG = control group; AE = aerobic exercises; RE = resistance exercise; MHR = maximum heart rate; wk = week; VF = visceral fat; TC = total cholesterol; GSIS = glucose-stimulated insulin secretion; BMI = body mass index; BW = body weight; WC = waist circumstance; IR = insulin resistance; HOMA-IR = insulin resistance index of homeostasis model assessment; ISI = insulin sensitivity index; FPG = fasting plasma glucose.
